# Perioperative Anemia Investigation and Management in Vascular Surgery: A Single-Center Retrospective Service Evaluation

**DOI:** 10.7759/cureus.114124

**Published:** 2026-08-07

**Authors:** Harun H Ali, Woolagasen Pillay

**Affiliations:** 1 Department of Vascular Surgery, Doncaster and Bassetlaw Teaching Hospitals NHS Foundation Trust, Doncaster, GBR

**Keywords:** blood transfusion, iron deficiency, perioperative anemia, service evaluation, vascular surgery

## Abstract

Background

Perioperative anemia is common in vascular surgery, but it is unclear how well national guidelines are followed in its investigation. This service evaluation examined how closely routine vascular surgical practice adhered to the British Society for Haematology (BSH) recommendations for investigating iron deficiency.

Methods

A retrospective service evaluation of consecutive adults undergoing vascular surgery at a single NHS center was performed. Electronic records were reviewed to determine the prevalence of preoperative anemia, adherence to the BSH recommendations for iron deficiency investigation, perioperative iron therapy, transfusion, and postoperative hemoglobin recovery. Exploratory comparisons were made by preoperative anemia status.

Results

Fifty-seven patients were included in the evaluation, and preoperative hemoglobin levels were documented for 53/57 patients (93.0%), of whom 32/53 (60.4%) were anemic (hemoglobin <130 g/L). Among patients with anemia, ferritin was measured in 14/32 (43.8%), but only 8/32 (25.0%) underwent a complete iron deficiency assessment. Iron therapy was initiated during the perioperative episode in 9/57 patients (15.8%). Nine patients (9/57, 15.8%) received a postoperative RBC transfusion, of whom 8/9 (88.9%) had preoperative anemia. Patients with preoperative anemia had a lower postoperative hemoglobin nadir than those without anemia (median 87 vs 126 g/L; Mann-Whitney U = 530.5; p < 0.001). Hemoglobin recovery was assessable in 35/57 patients (61.4%), of whom 22/35 (62.9%) had documented recovery to at least their preoperative concentration during available follow-up.

Conclusions

Preoperative anemia was common in this cohort, but investigation of its underlying cause was often incomplete. Only 8/32 patients with anemia (25.0%) underwent a complete iron deficiency assessment, hindering the classification of iron status and the provision of cause-directed management. Improving adherence to a structured diagnostic pathway is the principal opportunity for local quality improvement.

## Introduction

Perioperative anemia is common among patients undergoing vascular surgery and represents an important component of perioperative risk assessment [1-3]. In a UK National Vascular Registry analysis of 13,641 patients undergoing lower limb revascularization, 42.1% had preoperative anemia, which was independently associated with reduced one-year amputation-free survival and increased hospital readmission [1]. These associations have prompted interest in identifying and managing preoperative anemia, although evidence for the effect of specific corrective interventions remains uncertain [2-4]. Because perioperative anemia may have several underlying causes, including potentially reversible deficiencies, current national guidance recommends systematic investigation to establish the likely etiology and guide treatment [5].

Iron deficiency is one such potentially reversible cause of perioperative anemia. It may be absolute, reflecting depleted iron stores, or functional, in which iron availability is restricted despite apparently preserved stores, often in association with inflammation [5]. Ferritin is a useful marker of iron stores, but as an acute-phase reactant, it may be elevated in the presence of inflammation and therefore requires careful interpretation [5,6].

The 2024 British Society for Haematology (BSH) guideline recommends assessing ferritin in conjunction with transferrin saturation (TSAT) in patients with suspected iron deficiency and concomitant inflammation [5]. It also recommends considering non-iron causes of preoperative anemia, including vitamin B12 and folate deficiency, particularly where anemia is macrocytic or otherwise unexplained [5]. Without adequate investigation, iron status may remain unclassified, making cause-directed treatment difficult and potentially leading to unnecessary empirical iron therapy or failure to treat another reversible cause [5,6].

Although correction of iron deficiency may improve preoperative hemoglobin levels, evidence that iron treatment reduces transfusion requirements or postoperative complications is inconsistent [4,5]. Nevertheless, establishing the underlying cause remains an important component of patient blood management (PBM) because the appropriate treatment depends on the etiology [5,6]. Guidance on the investigation and management of perioperative anemia is now well established [5,6]. However, there is relatively little published evidence describing how closely routine vascular surgical practice adheres to these recommendations. The multicenter UK Cardiac and Vascular Surgery Interventional Anaemia Response (CAVIAR) study evaluated the implementation of a perioperative anemia pathway across cardiac and vascular surgery services and identified substantial barriers to successful implementation [7]. Routine investigation and management in the absence of such a pathway have been described less frequently.

This service evaluation assessed perioperative anemia investigation and management in routine vascular surgical practice against current BSH recommendations. The primary objectives were to describe the prevalence of preoperative anemia and completion of recommended iron deficiency investigations. Secondary outcomes included perioperative iron therapy, RBC transfusion, and postoperative hemoglobin recovery. Exploratory analyses compared investigation, management, and postoperative outcomes according to preoperative anemia status and examined factors associated with postoperative transfusion and documented hemoglobin recovery.

## Materials and methods

Study design and setting

This was a retrospective service evaluation conducted at Doncaster and Bassetlaw Teaching Hospitals NHS Foundation Trust (DBTH). Anemia investigation practices were evaluated against the BSH guideline recommendations. According to the UK Health Research Authority decision tool, this project met the criteria for a service evaluation rather than research. It was therefore registered as a service evaluation under the Trust’s clinical governance framework, and Research Ethics Committee approval was not required.

Patient selection and data collection

**Figure 1:**
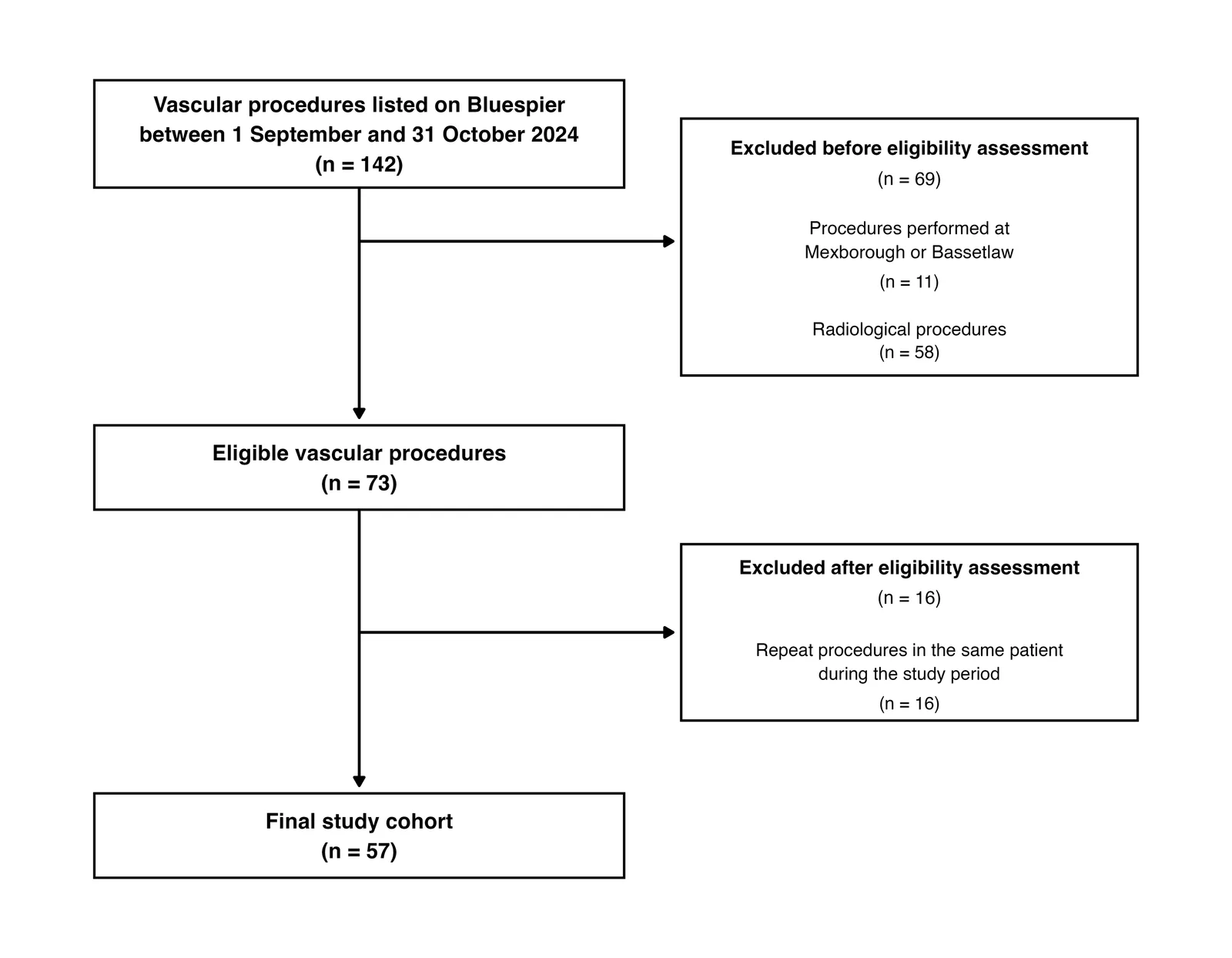
Identification and selection of the study cohort. Flow diagram showing identification of the study cohort from all vascular procedures recorded in Bluespier (Bluespier International, Droitwich, Worcestershire, UK), the theater scheduling system, between September 1, 2024 and October 31, 2024. Exclusion criteria were applied sequentially, resulting in a final cohort of 57 patients.

We included all consecutive adult patients undergoing vascular surgery at DBTH between September 1, 2024 and October 31, 2024. We excluded radiological procedures, procedures performed at satellite hospitals, and patients who underwent more than one procedure during the study period. The cohort selection process is summarized in Figure 1.

Clinical data were collected retrospectively from the electronic patient record using a standardized data collection proforma. Variables extracted included procedure type, preoperative hemoglobin concentration, day 1 postoperative hemoglobin concentration, postoperative hemoglobin nadir, ferritin measurement, completion of a BSH-recommended iron deficiency assessment, iron deficiency classification, vitamin B12 and folate assessment, perioperative iron therapy, and perioperative RBC transfusion. All available postoperative hemoglobin measurements and their dates were reviewed to assess hemoglobin recovery.

Definitions

Preoperative anemia was defined according to the BSH guidance as a hemoglobin concentration below 130 g/L. Absolute iron deficiency was defined as ferritin <30 µg/L and functional iron deficiency as ferritin 30-100 µg/L with TSAT <20%. A complete iron deficiency assessment was defined as a ferritin measurement with a TSAT measurement where ferritin was 30-100 µg/L. Patients without sufficient testing to determine iron status were classified as not assessable.

The postoperative hemoglobin nadir was the lowest hemoglobin concentration recorded during the postoperative inpatient stay. Hemoglobin recovery was defined as the first postoperative hemoglobin concentration equal to or greater than the preoperative value. Recovery time was calculated from the date of surgery to the first hemoglobin concentration meeting this criterion. Patients with postoperative follow-up measurements in whom no measurement reached the preoperative concentration were classified as having no documented recovery during available follow-up. Recovery was classified as not assessable when the preoperative hemoglobin concentration or postoperative follow-up measurements were unavailable.

Perioperative iron therapy included oral or intravenous iron administered before or after surgery, together with documentation of patients already receiving established iron supplementation before admission. Patients already receiving established iron therapy were distinguished from those in whom treatment was initiated before surgery or during the postoperative inpatient stay. RBC transfusion was categorized as occurring before or after surgery. Postoperative transfusion was defined as an RBC transfusion administered during the postoperative inpatient stay.

Outcome measures

The primary outcomes were the prevalence of preoperative anemia and completion of BSH-recommended iron deficiency investigations. Secondary descriptive outcomes included vitamin B12 and folate measurement, perioperative iron therapy, RBC transfusion, and postoperative hemoglobin decline and recovery.

Exploratory analyses compared investigation and management according to preoperative anemia status, including ferritin measurement, completion of iron deficiency assessment, vitamin B12 and folate measurement, and initiation of perioperative iron therapy. Postoperative hemoglobin nadir, perioperative hemoglobin decline, postoperative transfusion, and documented hemoglobin recovery were also compared according to preoperative anemia status.

An additional exploratory analysis compared preoperative hemoglobin concentration according to postoperative transfusion status. Among patients with assessable follow-up, preoperative hemoglobin concentration, preoperative anemia, perioperative hemoglobin decline, postoperative transfusion, and perioperative iron therapy were compared according to documented recovery status. These analyses were hypothesis-generating and were not intended to establish causal relationships.

Statistical analysis

Statistical analyses were performed using R (version 4.5.1; R Foundation for Statistical Computing, Vienna, Austria). Continuous variables were assessed for normality using the Shapiro-Wilk test and are presented as mean ± SD or median with IQR, as appropriate. Categorical variables are summarized as frequencies and percentages.

Exploratory comparisons between groups were performed using the Mann-Whitney U test for continuous variables and Fisher’s exact test for categorical variables because of the small sample size and expected cell counts. All statistical tests were two-sided, with statistical significance defined as p < 0.05. The Mann-Whitney U statistic is reported alongside the corresponding p-value. Fisher’s exact test does not produce an equivalent test statistic; therefore, only the p-value is reported for categorical comparisons. No adjustment was made for multiple comparisons because the analyses were exploratory. Missing data were not imputed, and analyses were performed using complete-case analysis for each variable.

## Results

Patient cohort

**Figure 2:**
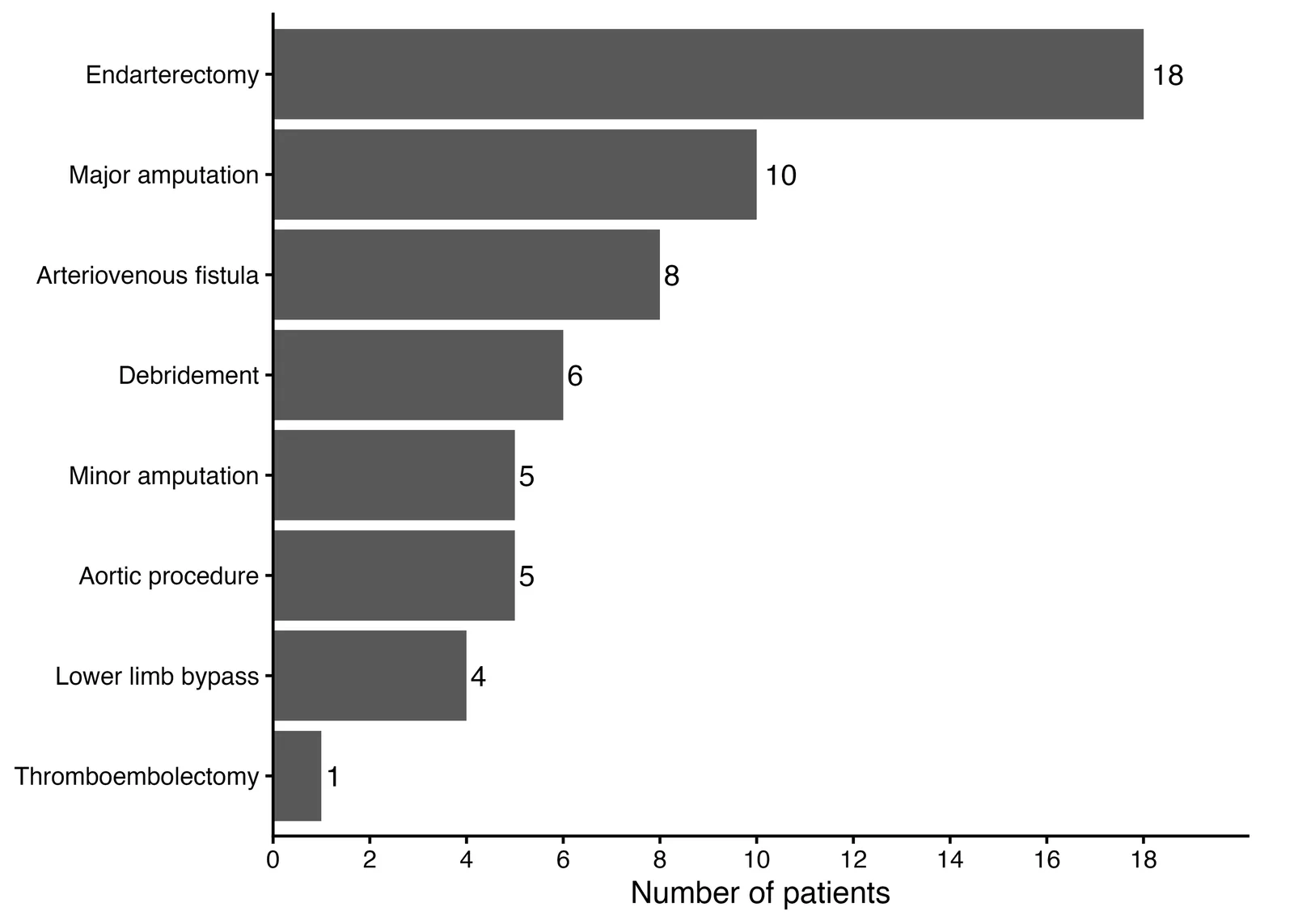
Distribution of vascular procedures within the study cohort. Horizontal bar chart illustrating the frequency of vascular procedures performed in the study cohort (total cohort = 57 patients).

A total of 57 patients met the inclusion criteria and were included in the evaluation. Procedure distribution and hemoglobin data availability are summarized in Table 1, while the distribution of vascular procedures is illustrated in Figure 2. Endarterectomy was the most common procedure (18/57, 31.6%), followed by major amputation (10/57, 17.5%) and arteriovenous fistula formation (8/57, 14.0%).

**Table 1:** Vascular procedure distribution and hemoglobin data availability in the study cohort. Data are presented as n (%).

Characteristic	Category	n (%)
Cohort	Total patients	57 (100.0)
Procedure type	Aortic procedure	5 (8.8)
Procedure type	Lower limb bypass	4 (7.0)
Procedure type	Debridement	6 (10.5)
Procedure type	Endarterectomy	18 (31.6)
Procedure type	Arteriovenous fistula	8 (14.0)
Procedure type	Major amputation	10 (17.5)
Procedure type	Minor amputation	5 (8.8)
Procedure type	Thromboembolectomy	1 (1.8)
Hemoglobin availability	Preoperative hemoglobin available	53 (93.0)
Hemoglobin availability	Day 1 postoperative hemoglobin available	53 (93.0)

Perioperative hemoglobin assessment

Preoperative and day 1 postoperative hemoglobin measurements were available for 53/57 patients (93.0%). Of these, 32/53 (60.4%) had preoperative anemia, and 21/53 (39.6%) did not. Mean hemoglobin decreased from 126.7 ± 19.4 g/L preoperatively to 111.6 ± 18.6 g/L on postoperative day 1.

**Figure 3:**
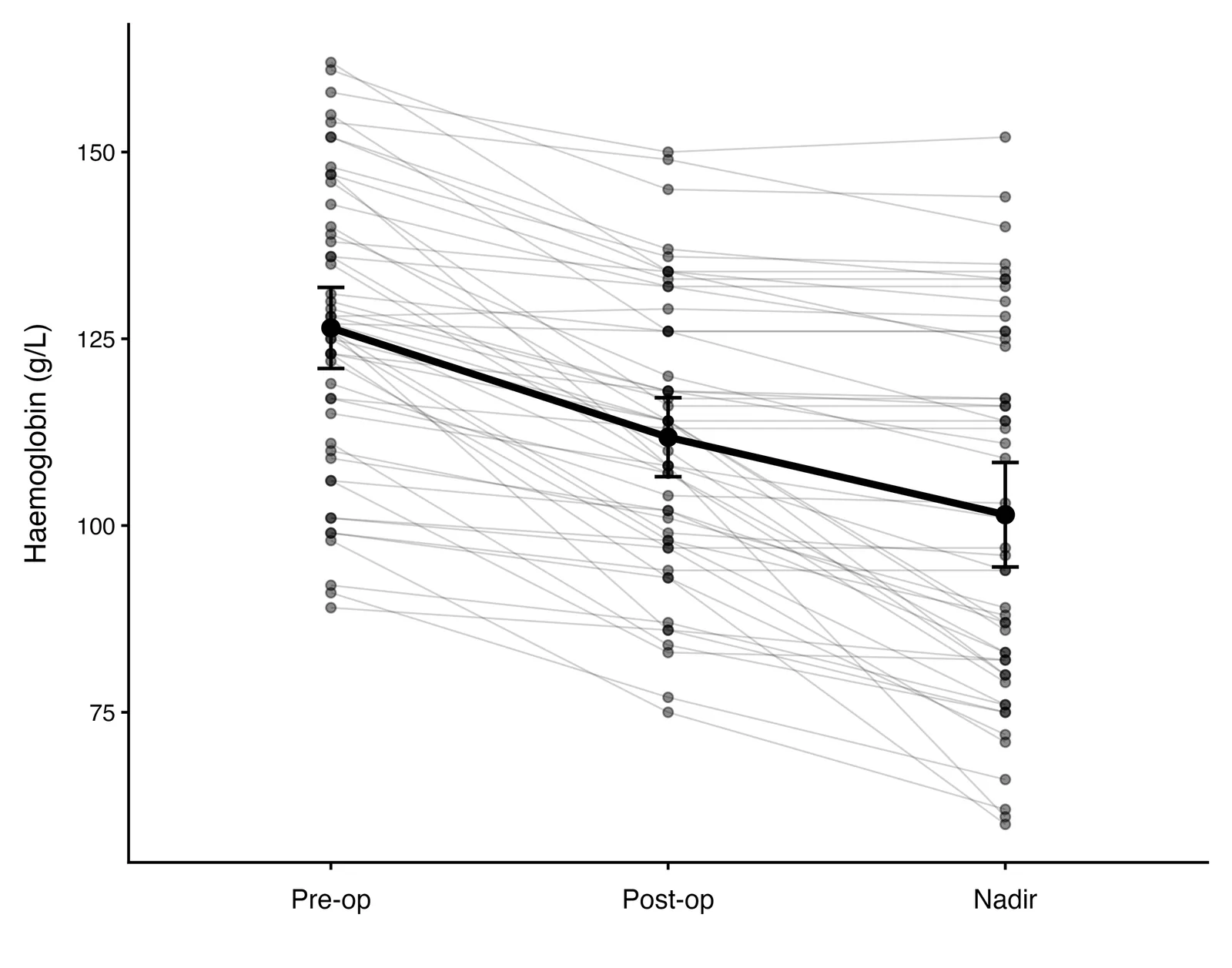
Perioperative hemoglobin trajectory following vascular surgery. Individual hemoglobin trajectories are shown for 51/57 patients (89.5%) with complete preoperative, day 1 postoperative, and postoperative nadir measurements. Thin lines connect measurements from the same patient. The thicker line and larger points represent cohort means, with error bars showing 95% CIs. Nadir = lowest hemoglobin concentration recorded during the postoperative inpatient stay

A postoperative hemoglobin nadir was available for 51/57 patients (89.5%). The mean nadir was 101.5 ± 24.9 g/L, and the mean decline from the preoperative concentration was 25.0 ± 16.7 g/L. The median decline was 22 g/L (IQR 13-36 g/L). Perioperative hemoglobin measurements are summarized in Table 2 and illustrated in Figure 3.

**Table 2:** Perioperative hemoglobin concentrations and decline from the preoperative value. Data are presented as mean (SD) and median (IQR). Hemoglobin concentrations are reported in g/L.

Measure	Available observations, n/N (%)	Mean (SD), g/L	Median (IQR), g/L
Preoperative hemoglobin	53/57 (93.0%)	126.7 (19.4)	127.0 (111.0-140.0)
Day 1 postoperative hemoglobin	53/57 (93.0%)	111.6 (18.6)	113.0 (98.0-126.0)
Postoperative hemoglobin nadir	51/57 (89.5%)	101.5 (24.9)	97.0 (81.0-124.5)
Decline from preoperative hemoglobin to postoperative nadir	51/57 (89.5%)	25.0 (16.7)	22.0 (13.0-36.0)

Investigation and management of perioperative anemia

Ferritin was measured in 15/57 patients (26.3%), and 8/57 (14.0%) underwent a complete iron deficiency assessment. Among the 32 patients with preoperative anemia, ferritin was measured in 14/32 (43.8%), and a complete assessment was performed in 8/32 (25.0%). Both ferritin measurement and completion of iron deficiency assessment were more frequent among patients with preoperative anemia than among those without anemia (14/32 (43.8%) vs 1/21 (4.8%), Fisher’s exact test, p = 0.002; and 8/32 (25.0%) vs 0/21 (0%), Fisher’s exact test, p = 0.016, respectively).

Among the eight patients with a complete assessment, 6/8 (75.0%) had no evidence of iron deficiency, and 2/8 (25.0%) met the prespecified criteria for functional iron deficiency. No patients (0/8, 0%) met the criteria for absolute iron deficiency. Iron status was not assessable in 49/57 patients (86.0%) because of incomplete investigation.

**Figure 4:**
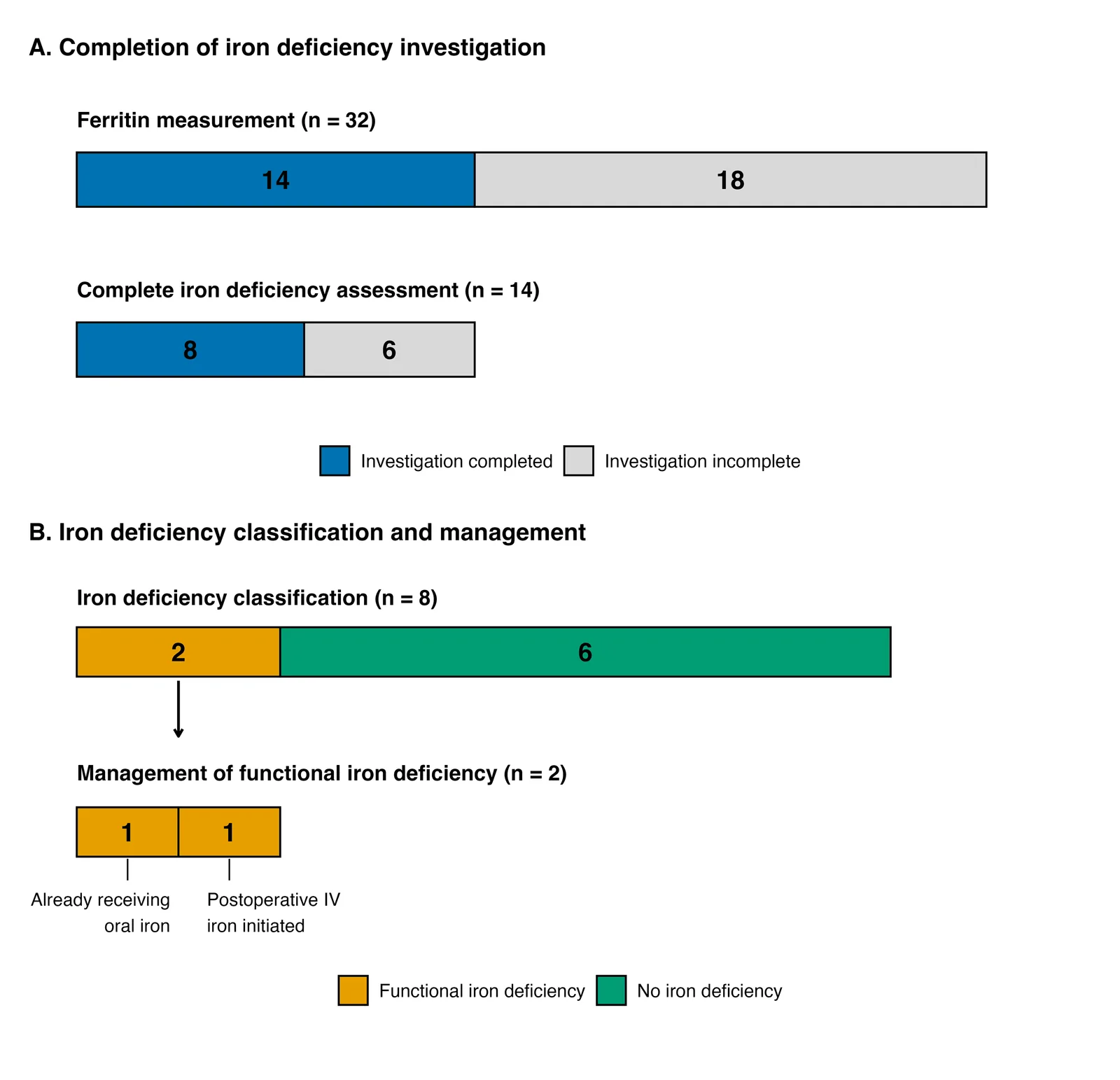
Investigation, classification, and management of iron deficiency among patients with preoperative anemia. (A) Completion of iron deficiency investigation among the 32 patients with preoperative anemia. Bar widths are proportional to the number of patients entering each stage, and segment widths indicate whether investigation was completed. (B) Iron deficiency classification among the eight patients who completed assessment, followed by management of the two patients with functional iron deficiency. Numbers within bars represent patient counts.

Vitamin B12 and folate were measured in 6/57 patients (10.5%), all of whom had preoperative anemia. Five of the six patients (83.3%) had normal results, and one (16.7%) had folate deficiency. Testing was more frequent among patients with preoperative anemia, although the difference was not statistically significant (6/32 (18.8%) vs 0/21 (0%), Fisher’s exact test, p = 0.070). Investigation and management are summarized in Table 3. Figure 4A shows completion of iron deficiency investigation, while Figure 4B shows subsequent classification and management.

**Table 3:** Investigation and management of perioperative anemia according to preoperative anemia status. Data are presented as n/N (%). Anemia-stratified analyses include the 53 patients with an available preoperative hemoglobin measurement. Comparisons were performed using Fisher’s exact test. A p-value <0.05 was considered statistically significant.

Measure	Overall, n/N (%)	Preoperative anemia, n/N (%)	No preoperative anemia, n/N (%)	p-value
Ferritin measured	15/57 (26.3%)	14/32 (43.8%)	1/21 (4.8%)	0.002
Complete iron deficiency assessment	8/57 (14.0%)	8/32 (25.0%)	0/21 (0%)	0.016
Vitamin B12/folate measured	6/57 (10.5%)	6/32 (18.8%)	0/21 (0%)	0.07
Iron initiated during perioperative episode	9/57 (15.8%)	9/32 (28.1%)	0/21 (0%)	0.008

Iron therapy was documented in 14/57 patients (24.6%). Five of these patients (5/14, 35.7%) were already receiving oral iron before admission, while treatment was initiated during the perioperative episode in 9/57 patients (15.8%): 2/9 (22.2%) before surgery and 7/9 (77.8%) postoperatively. Treatment was initiated in 9/32 patients with preoperative anemia (28.1%), compared with 0/21 without anemia (0%; Fisher’s exact test, p = 0.008). Oral iron was the predominant treatment, with intravenous iron administered to one patient. Of the two patients meeting the criteria for functional iron deficiency, 1/2 (50.0%) was already receiving oral iron and 1/2 (50.0%) received postoperative intravenous iron.

Blood transfusion and hemoglobin recovery

Overall, 10/57 patients (17.5%) received an RBC transfusion. One patient (1/57, 1.8%) was transfused preoperatively, and 9/57 (15.8%) received a postoperative transfusion. Of those transfused postoperatively, 8/9 (88.9%) had preoperative anemia. Among all nine patients receiving a postoperative transfusion, the mean postoperative hemoglobin nadir was 69.9 ± 8.2 g/L, with a median of 71 g/L (IQR 62-75 g/L).

Follow-up was sufficient to assess hemoglobin recovery in 35/57 patients (61.4%). Hemoglobin reached the preoperative value in 22/35 patients (62.9%) after a median of 16 days (IQR 6-27; range 0-84 days). Of the 22 patients whose recovery could not be assessed, 18/22 (81.8%) lacked a follow-up hemoglobin measurement, and 4/22 (18.2%) had incomplete perioperative measurements.

Exploratory comparative analyses

Patients with preoperative anemia had a lower postoperative hemoglobin nadir than those without anemia (median 87 vs 126 g/L; Mann-Whitney U test, U = 530.5, p < 0.001), while perioperative hemoglobin decline was similar between the groups (median 23 vs 18 g/L; Mann-Whitney U test, U = 288.5, p = 0.685). Postoperative transfusion occurred in 8/32 patients with preoperative anemia (25.0%) and 1/21 without anemia (4.8%; Fisher’s exact test, p = 0.071). Documented recovery occurred in 16/23 patients with preoperative anemia (69.6%) and 6/12 without anemia (50.0%; Fisher’s exact test, p = 0.292). These comparisons are summarized in Table 4.

**Table 4:** Postoperative outcomes according to preoperative anemia status. Data are presented as median (IQR) or n/N (%). Continuous variables were compared using the Mann-Whitney U test, and categorical variables were compared using Fisher’s exact test. Hemoglobin recovery analysis included only patients with sufficient follow-up measurements to assess recovery. A p-value <0.05 was considered statistically significant.

Outcome	No preoperative anemia (n = 21)	Preoperative anemia (n = 32)	Test statistic	p-value
Lowest postoperative hemoglobin, median (IQR), g/L	126 (114-134)	87 (76-100)	U = 530.5	<0.001
Perioperative hemoglobin decline, median (IQR), g/L	18 (13-30)	23 (13-37)	U = 288.5	0.685
Postoperative transfusion, n/N (%)	1/21 (4.8%)	8/32 (25.0%)	Not applicable	0.071
Hemoglobin recovery, n/N (%)	6/12 (50.0%)	16/23 (69.6%)	Not applicable	0.292

Patients who received a postoperative transfusion had a lower preoperative hemoglobin concentration than those who did not (median 106 g/L, IQR 98-123, n = 9, vs 128 g/L, IQR 117-143.8, n = 44; Mann-Whitney U test, U = 312, p = 0.007). Patients without documented hemoglobin recovery had a greater perioperative hemoglobin decline than those with documented recovery (median 37 g/L, IQR 21-44, n = 13, vs 13 g/L, IQR 11-21.3, n = 22; Mann-Whitney U test, U = 44, p < 0.001). Preoperative hemoglobin did not differ significantly between patients without and with documented recovery (median 127 g/L, IQR 125-140, vs 125 g/L, IQR 102.3-129.8; Mann-Whitney U test, U = 104, p = 0.188). There were no statistically significant differences in documented recovery according to postoperative transfusion (4/7 (57.1%) vs 18/28 (64.3%); Fisher’s exact test, p = 1.000) or recorded perioperative iron exposure (7/10 (70.0%) vs 15/25 (60.0%); Fisher’s exact test, p = 0.709).

## Discussion

Preoperative anemia was very common, affecting 32/53 patients with hemoglobin data (60.4%). However, only 8/32 anemic patients (25.0%) underwent the iron studies recommended by the BSH, leaving iron status undetermined in the majority.

The prevalence of preoperative anemia in our cohort (32/53, 60.4%) was higher than the 42.1% reported by Birmpili et al. in the UK National Vascular Registry [1]. Other vascular cohorts have reported prevalence estimates of 52.3% among patients undergoing revascularization for chronic limb-threatening ischemia [8] and 70% among those undergoing infrainguinal bypass for chronic limb-threatening ischemia [9]. Our estimate therefore lies within the range reported in vascular surgical populations, although differences in case mix and definitions of anemia limit direct comparison. The high prevalence is clinically relevant given the reported associations between preoperative anemia and postoperative morbidity, mortality, and transfusion [2,3].

Incomplete testing meant that iron deficiency could not be distinguished reliably from other causes of anemia. The BSH guideline recommends using ferritin with TSAT where ferritin may be influenced by inflammation and considering other causes when iron deficiency is not identified [5]. Similar principles are included in the International Consensus Conference on Anemia Management in Surgical Patients, which emphasizes diagnosis and treatment directed at the underlying cause [6]. The low number of patients classified as iron deficient should therefore not be interpreted as evidence that iron deficiency was uncommon in this cohort.

The findings are relevant to the development of a structured perioperative anemia pathway. PBM is a multidisciplinary approach that aims to optimize patient outcomes through optimization of anemia management, minimization of blood loss, and appropriate use of transfusion [6]. The multicenter CAVIAR study identified practical barriers to implementing perioperative anemia pathways across vascular and cardiac surgical services [7]. Our evaluation provides a baseline against which changes in local investigation and management can be assessed after pathway implementation.

Iron therapy was initiated in 9/57 patients (15.8%), all of whom had preoperative anemia. However, incomplete investigation often made it difficult to determine whether treatment was indicated. The treatment data should therefore not be interpreted as evidence of either undertreatment or appropriate treatment. Although iron therapy may increase hemoglobin concentration, evidence that it reduces transfusions or major postoperative complications remains inconsistent [4,10]. These findings reinforce the principles of PBM, which emphasize cause-directed treatment of anemia rather than empirical iron therapy. More broadly, implementation of PBM programs has been associated with reduced blood transfusion without compromising patient safety [6,11].

Patients with preoperative anemia had lower postoperative hemoglobin nadirs than those without anemia, although the decline from the preoperative value was similar between groups. This suggests that the difference in postoperative concentrations largely reflected their lower starting hemoglobin. Hemoglobin decline cannot, however, be interpreted as a direct measure of operative blood loss because it may also be affected by fluid administration, transfusion, and the timing of blood sampling.

Postoperative transfusion was more frequent among patients with preoperative anemia (8/32, 25.0%) than among those without anemia (1/21, 4.8%), although the difference did not reach statistical significance. Patients who received a postoperative transfusion also had lower preoperative hemoglobin concentrations. These findings are consistent with previous observational evidence linking preoperative anemia with transfusion [2,3], but the small number of transfusions limits interpretation.

The median hemoglobin nadir among patients who received a postoperative transfusion was 71 g/L, close to the 70 g/L threshold at which restrictive transfusion guidance recommends considering transfusion for most hemodynamically stable adults [12]. The guidance also emphasizes clinical context and allows higher thresholds for some patients with cardiovascular disease or undergoing particular surgical procedures [12]. As transfusions followed the recorded nadir, the low hemoglobin concentration was likely to have contributed to the decision to transfuse. However, immediate pretransfusion measurements and clinical indications were not consistently documented, so adherence to transfusion guidance could not be determined.

Among patients with sufficient follow-up, greater perioperative hemoglobin decline was associated with a lower likelihood of documented recovery to the preoperative concentration. No statistically significant differences in recovery were found according to preoperative anemia, postoperative transfusion, or perioperative iron exposure. These comparisons were based on small groups and variable follow-up. Iron exposure also included treatments that differed in timing, route, and indication, so the findings cannot establish whether iron therapy influenced recovery.

This evaluation has several limitations. It was conducted at a single center over two months and included a small, heterogeneous group of vascular procedures. As a retrospective review, the study relied on the completeness and accuracy of routinely recorded clinical data and was susceptible to information bias and residual confounding. Patients undergoing multiple procedures during the evaluation period were excluded, which may have removed patients with more complex clinical courses. Iron status could not be classified in most patients because testing was incomplete. A contemporaneous inflammatory marker was not available alongside the iron studies. Although the underlying vascular pathology and need for surgery made concomitant inflammation clinically plausible, it was not confirmed biochemically in the two patients classified as having functional iron deficiency. Their classification was therefore based on the prespecified ferritin and TSAT criteria alone. Hemoglobin recovery could be assessed in only 35/57 patients (61.4%), and follow-up blood tests were performed at different intervals. Recovery after the final available measurement may therefore have been missed. The exploratory analyses were limited by small complete-case samples, multiple unadjusted comparisons, and only 9/57 postoperative transfusions (15.8%). Iron treatment also varied in timing, route, and indication, limiting interpretation of its relationship with recovery. Finally, inconsistent documentation of immediate pretransfusion hemoglobin concentrations and clinical indications prevented formal assessment of transfusion practice against guideline recommendations.

## Conclusions

Preoperative anemia was common in this vascular surgical cohort, affecting 32/53 patients with an available preoperative hemoglobin measurement (60.4%). However, only 8/32 patients with anemia (25.0%) underwent a complete iron deficiency assessment, leaving iron status unclassified in most cases. The main gap in current practice was therefore not the failure to identify anemia but the failure to complete the investigation needed to establish iron status. Addressing this gap would allow perioperative treatment to be better matched to the individual patient.
